# Lung epithelial GM-CSF improves host defense function and epithelial repair in influenza virus pneumonia—a new therapeutic strategy?

**DOI:** 10.1186/s40348-016-0055-5

**Published:** 2016-08-01

**Authors:** Barbara Rösler, Susanne Herold

**Affiliations:** 1 Dr von Hauner Children’s Hospital, Ludwig-Maximilians-University, Lindwurmstrasse 4, 80337 Munich, Germany; 2 Comprehensive Pneumology Center Munich (CPC-M), Member of the German Center for Lung Research (DZL), Munich, Germany; 3 Department of Internal Medicine II, Section for Infectious Diseases, University Hospital Giessen and Marburg, Klinikstr. 33, Giessen, 35392 Germany; 4 Universities Giessen and Marburg Lung Center (UGMLC), Member of the German Center for Lung Research (DZL), Giessen, Germany; 5 Department of Internal Medicine II, Section for Infectious Diseases, Universities Giessen and Marburg Lung Center (UGMLC), Klinikstr. 33, 35392 Giessen, Germany

**Keywords:** Influenza, Pneumonia, ARDS, GM-CSF, Therapy, AEC

## Abstract

Influenza viruses (IVs) circulate seasonally and are a common cause of respiratory infections in pediatric and adult patients. Additionally, recurrent pandemics cause massive morbidity and mortality worldwide. Infection may result in rapid progressive viral pneumonia with fatal outcome. Since accurate treatment strategies are still missing, research refocuses attention to lung pathology and cellular crosstalk to develop new therapeutic options.

Alveolar epithelial cells (AECs) play an important role in orchestrating the pulmonary antiviral host response. After IV infection they release a cascade of immune mediators, one of which is granulocyte and macrophage colony-stimulating factor (GM-CSF). GM-CSF is known to promote differentiation, activation and mobilization of myeloid cells. In the lung, GM-CSF drives immune functions of alveolar macrophages and dendritic cells (DCs) and also improves epithelial repair processes through direct interaction with AECs. During IV infection, AEC-derived GM-CSF shows a lung-protective effect that is also present after local GM-CSF application. This mini-review provides an overview on GM-CSF-modulated immune responses to IV pneumonia and its therapeutic potential in severe IV pneumonia.

## Introduction

Respiratory viral infections are often seen in pediatric patients [1]. They may cause primary viral pneumonia that may progress to lung failure with fatal outcome. Apart from respiratory syncytial virus (RSV) and other respiratory viruses, influenza virus (IV) infection is a common cause of acute respiratory failure on pediatric intensive care units (ICUs) [2].

IVs are enveloped single-stranded negative-sensed RNA viruses, divided into three genera A, B, and C [3]. They cause respiratory infections in humans and not only occur seasonally but also occur in recurrent pandemics [4]. IV A is further divided into subtypes that differ in the surface glycoproteins hemagglutinin (HA) and neuraminidase (NA). The viral HA serves to attach to host cells; the NA protein cares for viral release after replication [5]. Due to the segmented nature of its genome and a high rate of mutations during replication, seasonal IVs show an annual change of antigenic qualities in the HA and NA genes, requiring annual immunization. Mutations in the coding genes for HA and NA are called *antigenic drift*, while the reassortment of viral gene segments is called *antigenic shift* [3]. The creation of new viral subtypes by reassortment is only known to be prevalent in type A IVs with a large reservoir of different strains in animals, particularly waterfowl but also in other birds and mammals [6].

Primary target cells for IV infection in humans are respiratory epithelial cells [7]. The current opinion is that the major route of infection proceeds by viral HA binding to sialic acid receptors on the epithelial cells, followed by internalization via endocytosis [8]. The viral genome is replicated in the nucleus and translated in the cytoplasm, and mature virus particles are released from the cell via budding [5].

As soon as the infection spreads from the upper to the lower respiratory tract, alveolar epithelial cells (AECs) become primary targets for productive IV replication [4]. Pro-inflammatory mechanisms—together with a direct viral cytopathogenic effect—lead to AEC apoptosis. Additionally, lung pathology is characterized by loss of alveolar barrier function and alveolar edema fluid accumulation [9]. Ongoing inflammation leads to increased capillary/alveolar leakage, followed by severe hypoxemia [4] and results in acute respiratory distress syndrome in children (PARDS) and adults [10].

Rapid and effective viral clearance from the distal lung by immune effector cells and the initiation of epithelial repair processes including expansion of local epithelial progenitor cells and resealing of the epithelial layer are crucial to recover from IV-induced lung injury [11, 12]. The inflammatory immune response needs to be balanced between the elimination of virus and immune-mediated pulmonary injury to limit the damage to the respiratory tract [13].

Myeloid cells like monocytes, macrophages, dendritic cells (DCs), and their common precursor cells, summarized as mononuclear phagocytes, are crucial in driving IV clearance [12]. Combining sensor and effector functions of innate immunity, the lung epithelium plays an important role in coordinating, maintaining, and balancing the phagocyte-mediated antiviral host response [7, 14, 15]. The intimate spatial proximity of alveolar macrophages and the tissue-resident DC network with the distal lung epithelium provides an ideal basis for direct cell-cell communication. Mechanisms involved in this cellular crosstalk might represent potential targets for treatment.

### Pulmonary GM-CSF and its secretion during IV infection

The growth factor granulocyte and macrophage colony-stimulating factor (GM-CSF) is widely recognized to promote proliferation, differentiation, and activation of monocytes, granulocytes, macrophages, and DCs in vivo and plays an important role in immunity [16, 17]. Involved in proinflammatory cytokine response, it is discussed to be responsible for immunopathology in several inflammatory or autoimmune diseases [16]. It controls nonlymphoid tissue DC homeostasis [18] and was currently reported to modulate the development of inflammatory macrophages and monocytes [19]. In the lung, GM-CSF was shown not only to play a role in allergic airway disease [20, 21] but also to be crucial for antimicrobial pulmonary host defense function [22–24]. Additionally, it is essential for surfactant homeostasis. GM-CSF-deficient mice develop a similar pathology to human pulmonary alveolar proteinosis (PAP) [25]. An exceeded accumulation of surfactant phospholipids and proteins in the alveolar lining fluid impairs gas exchange and patients show increased susceptibility for microbial infections [22]. PAP is found to be associated with GM-CSF or GM-CSF receptor auto-antibodies or dysfunction [26].

Pulmonary GM-CSF is mainly expressed by AEC type II and released under inflammatory conditions [27]. Other cell types like macrophages, endothelial cells, fibroblasts and T cells also produce GM-CSF, but AECs were shown to be the only CD45^−^ cell population in the distal lung parenchyma that upregulates GM-CSF upon IV infection, and produces high levels of GM-CSF in the alveolar lining fluid [28]. New findings report AEC GM-CSF secretion in IV infection to be mediated through HGF/c-Met and TGF-α/epithelial growth factor receptor (EGFR) signaling [29].

### AEC-derived GM-CSF is highly protective in IV pneumonia

It is well established that AEC-released GM-CSF improves innate immune responses of myeloid cells, in particular alveolar macrophages. Alveolar macrophages of GM-CSF-deficient mice show abnormalities in morphology, maturation, and function, depending on transcription factor PU.1 [22, 26]. Sever-Chroneos et al. [30] found a decreased resistance to IV in GM-CSF-deficient mice due to impaired pathogen clearance by macrophages. This is stressed by findings from Berclaz et al. who demonstrated that the Fcɣ receptor (FcɣR)-mediated opsonophagocytosis of invaded pathogens by alveolar macrophages is highly dependent on GM-CSF signaling via PU.1 [31]. T cell-produced interferon ɣ (IFN ɣ) during course of infection also effected augmented FcɣR levels on alveolar macrophages which in turn stimulated IFN ɣ production by secretion of inflammatory cytokines (interleukin (IL)-18, IL-12), linking innate and adaptive immunity in a positive feedback loop. Elevated alveolar GM-CSF level in transgenic mice increased numbers and resistance of alveolar macrophages and provided protection against lethal IV infections [32]. Subramaniam et al. [33] observed a GM-CSF-dependent protection through stimulation of reactive oxygen species (ROS) production by macrophages not only against IV pneumonia itself but also against secondary bacterial pneumonia, a major cause of morbidity and mortality after IV infection. However, GM-CSF-dependent activation of pulmonary innate immunity does not explain the beneficial effects of AEC GM-CSF in IV infection in total. Lung CD103^+^ DCs were found to be key players for the GM-CSF-dependent lung protective effect by our group [28]. After IV infection, pulmonary CD103^+^ DCs are expanded and activated and their migration and antigen (Ag) presentation to the draining mediastinal lymph nodes is mediated by GM-CSF. This is associated with a better viral clearance and Ag-specific T cell recruitment, suggesting improved resident lung DC host defense capacities during IV infection by AEC GM-CSF. Min et al. [34] identified GM-CSF to be a major licensing factor of CD8^+^ T lymphocytes to activate DCs during priming in lymphoid tissue, providing a positive feedback loop in the stimulation of CD8^+^ T cell proliferation. Accordingly, Greter et al. [18] revealed GM-CSF to be indispensable for the induction of specific CD8^+^ T cell immunity. Chen et al. [35] also found GM-CSF to promote T cell, B cell, and DC maturation in order to enable the production of IV specific antibodies.

During IV pneumonia, GM-CSF furthermore has a lung barrier-protective effect and improves survival, also after local application [28, 30, 32, 36]. AEC-expressed GM-CSF has direct beneficial effects on the injured epithelium. It is crucial in mediating epithelial proliferation in inflammatory or hyperoxic lung injury, supports repair and restoration of barrier function, and induces the return to tissue homeostasis [27, 37]. In a bleomycin rat model, it was shown that defects in AEC II GM-CSF secretion are crucial for pathogenesis of pulmonary fibrosis [38]. Sturrock et al. displayed certain microRNAs (miRNAs) (133a and 133b) to suppress protective AEC GM-CSF secretion by inhibition of GM-CSF messenger RNA (mRNA) translation in oxidative stress [39]. This effect seemed to be AEC specific as the same miRNAs did not suppress T cell GM-CSF expression during hyperoxia.

**Fig. 1:**
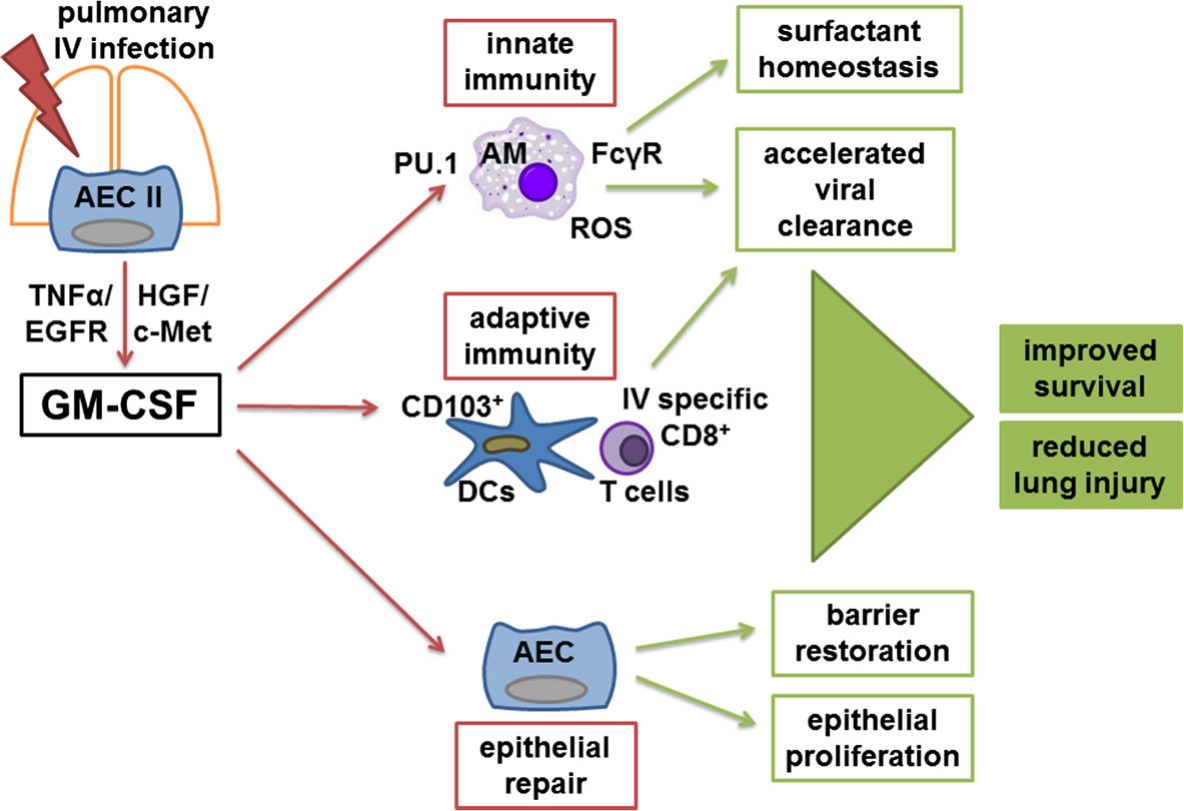
GM-CSF-modulated immune response to IV infection. After pulmonary IV infection GM-CSF is released from AEC II, mediated through HGF/c-Met and TGF-α/EGFR signaling. In an autocrine manner, it stimulates epithelial repair, including epithelial proliferation and barrier restoration. Innate and adaptive immunity are activated, resulting in accelerated viral clearance. Via PU.1, GM-CSF improves AM resistance, maturation, ROS production, and phagocytosis capacity, e.g., by the FcγR-mediated opsonophagocytosis. GM-CSF also stimulates activation and proliferation of DCs, especially CD103^+^ DCs, and T cells and enhances Ag priming and IV-specific CD8^+^ T cell recruitment. Altogether AEC GM-CSF leads to increased survival and reduced lung injury. *AEC* alveolar epithelial cells, *Ag* antigen, *AM* alveolar macrophage, *c-Met* hepatocyte growth factor receptor, *DC* dendritic cell, *EGFR* epithelial growth factor receptor, *FcγR* Fcɣ receptor, *GM-CSF* granulocyte and macrophage colony stimulating factor, *HGF* hepatocyte growth factor, *PU.1* transcription factor PU.1, *ROS* reactive oxygen species, *TGF-α* transcriptional growth factor α

Figure 1 summarizes reported findings on GM-CSF-modulated immune functions in IV pneumonia.

In adult patients with acute respiratory distress syndrome (ARDS), elevated GM-CSF levels in the bronchoalveolar lavage fluid (BALF) were shown to be associated with antiapoptotic effects and with improved epithelial barrier integrity and survival [40, 41]. In human lung sections, GM-CSF expressed by hyperplastic type II AECs mediates accumulation of neighboring CD1a^+^ DCs in inflamed lungs [42]. These findings suggest that GM-CSF is similarly beneficial and operative in humans. Of note, high serum cytokine levels of GM-CSF in pediatric intensive care unit (ICU) patients with severe influenza infection were associated with innate immune suppression and mortality [43], highlighting the particular beneficial effect of alveolar as opposed to circulating GM-CSF.

### GM-CSF as a therapeutic strategy

Current treatment strategies for IV infection focus on vaccines and antiviral agents. Due to its rapid genetic modification capacity, it will be a challenge to keep up with therapeutic targets as resistance rapidly occurs against current antivirals. Immune mediators that drive pulmonary host defense function like GM-CSF propose appealing alternative treatment strategies, especially if they do not only protect against IV infection itself but also against common complications like secondary bacterial pneumonia and severe damage of alveolar epithelium.

A clinical trial for low-dose intravenous GM-CSF treatment in adult patients with severe sepsis and respiratory dysfunction led to the conclusion that GM-CSF treatment was associated with improved gas exchange and might play a homeostatic role [44]. Another trial in adult patients with acute lung injury (ALI) failed to improve survival and ventilation parameters by intravenous GM-CSF application [45]. Regarding the mouse model, a high local concentration of GM-CSF in the alveolar lining fluid seems to be more promising than systemic application and also prevents systemic side effects. Alveolar GM-CSF is required in a distinct local quantity to balance pathogen clearance and immunopathology. This is why GM-CSF treatment should preferentially be investigated via the inhaled route in contrast to systemic application. Inflammation itself leads to compromised lung barrier integrity, causing systemic loss of locally delivered GM-CSF. Current strategies in the mouse model focus on GM-CSF conjugation to enhance bioavailability, prolong half-life, and reduce systemic side-effects of administered GM-CSF [46].

For patients with PAP, inhaled GM-CSF therapy displayed encouraging results [47, 48]. With regard to pneumonia-associated ARDS an off-label treatment with inhaled GM-CSF revealed an improvement in oxygenation and morbidity [49]. GM-CSF treatment increased activation of alveolar macrophages, important for host defense function. A multicenter, double-blind, placebo-controlled, randomized clinical trial on the efficacy of inhaled GM-CSF in adult patients with pneumonia-associated ARDS (GI-HOPE) was recently started within the nationwide German Center for Lung Research (NCT02595060). Similarly, an interventional safety/efficacy study for inhaled GM-CSF treatment in children with respiratory virus-associated ARDS has been implemented (iGRASP, NCT02601365), highlighting the strong potential of this drug in both adults and children suffering from severe viral pneumonia and related ARDS.

This review focuses on GM-CSF-mediated effects in IV pneumonia and associated ARDS. The clinical trials mentioned above investigate GM-CSF treatment not exclusively in IV pneumonia but in (viral) pneumonia-associated ARDS in general. While in the mouse model, specific conditions can be studied in detail, clinical conditions are different. In the clinical setting, the underlying germ of infection is not always clearly defined when patients present with pneumonia/ARDS and therapy needs to be initiated. Furthermore, bacterial superinfection of primary viral pneumonia is a common complication. GM-CSF-mediated protective effects are not limited to IV infection as they result in improvement of host defense capacities and epithelial repair in general, both critical processes in ARDS patients. GM-CSF might be working similarly in other respiratory viral infections as well. It was reported to prevent RSV-exacerbated airway hyperresponsiveness by alveolar macrophage maturation [50], for example. Protective effects of GM-CSF were also shown for bacterial pneumonia [23, 51, 52]. For these reasons, clinical studies focus on GM-CSF treatment on patients, who present with pneumonia-associated ARDS rather than exclusively IV pneumonia.

## Conclusion

Local therapeutic application of GM-CSF increases mononuclear phagocyte-mediated innate and adaptive host defense and accelerates epithelial repair processes during severe IV pneumonia in pre-clinical models. There is evidence that it might be a powerful therapy in viral (and bacterial) pneumonia and associated ARDS and eventually even other forms of ARDS in children and adults. Current data suggest that local application by the inhalative route seems to be most promising.

## Abbreviations

AEC, alveolar epithelial cell; Ag, antigen; ALI, acute lung injury; AM, alveolar macrophage; ARDS, acute respiratory distress syndrome; BALF, bronchoalveolar lavage fluid; c-Met, hepatocyte growth factor receptor; DC, dendritic cell; EGFR, epithelial growth factor receptor; FcɣR, Fcɣ receptor; GI-HOPE, clinical trial on GM-CSF treatment in ARDS patients; GM-CSF, granulocyte and macrophage colony stimulating factor; HA, hemagglutinin; HGF, hepatocyte growth factor; ICU, intensive care unit; IFNγ, interferon-γ; iGRASP, clinical trial on GM-CSF treatment in children with virus-associated ARDS; IL, interleukin; IV, influenza virus; miRNA, microRNA; mRNA, messenger RNA; NA, neuraminidase; PAP, pulmonary alveolar proteinosis; PARDS, pediatric acute respiratory distress syndrome; PU.1, transcription factor PU.1; ROS, reactive oxygen species; RSV, respiratory syncytial virus; TGF α, transcriptional growth factor α
